# Home vision therapy and prism prescription in presbyopic persons with convergence insufficiency: study protocol for a randomized controlled trial

**DOI:** 10.1186/s12886-024-03411-y

**Published:** 2024-04-15

**Authors:** Saeid Abdi, Haleh Kangari, Saeed Rahmani, Alireza Akbarzadeh Baghban, Zahra Kamary Rad

**Affiliations:** 1 Department of Optometry, Faculty of Rehabilitation, Shahid Beheshti University of Medical Sciences, Tehran, Iran; 2 Proteomics Research Center, Department of Biostatistics, School of Allied Medical Sciences, Shahid Beheshti University of Medical Sciences, Tehran, Iran

**Keywords:** Convergence insufficiency, Presbyopia, Home vision therapy, Prism prescription

## Abstract

**Background:**

Convergence insufficiency is a common issue in the field of binocular vision. Various treatment options have been suggested for managing this condition, but their efficacy in individuals with presbyopia remains unclear. The objective of this study is to compare the effectiveness of home-based vision therapy and prism prescription, in presbyopic patients with convergence insufficiency.

**Methods/design:**

It is a randomized, prospective, double-blind clinical trial, with total of 150 participants randomly assigned to the three groups. The Control Group will receive a new near glasses as a conventional prescription, along with aimless and random eye movement exercises that do not have any convergence or accommodation effects. The Home Vision Therapy Group will receive new near glasses with accommodative and convergence eye exercises. The Prism Group will receive a near prismatic glasses prescribed using the Sheard’s criterion. All treatments will be administered for a period of 2 months, and measurements of the modified convergence insufficiency symptoms survey (CISS), near point convergence, near phoria, and positive fusional vergence will be taken at baseline, one month later, and at the end of the treatment.

**Discussion:**

We aim to identify which component - either the prism prescription or the home vision therapy - is more effective in improving binocular abilities and reducing patients’ symptom scores.

**Trial registration:**

ClinicalTrials.gov NCT05311917 with last update on 04/22/2023.

## Background

Convergence insufficiency (CI) is a binocular vision problem characterized by an excess of exophoria in near activities compared to distance, moreover, its includes remoted near point of convergence (NPC) and decreased positive fusional vergence (PFV) [1]. Its prevalence typically ranges from 2.25 to 17.6%, depending on the population studied and the criteria used to define it [2–5]. This problem is also frequently observed in individuals with presbyopia with prevalence of 29.6% [4]. The most common ocular finding in these patients is an increase in near exophoria, which can impair performance in activities such as reading, computer work, and other near tasks [6]. Symptoms of CI include difficulty seeing at close range, headaches, eye pain during reading or studying, blurred vision, diplopia, movement of words while reading, a feeling of pressure in the eyes, and difficulty concentrating. Signs of it include an increasing near point of convergence, more exophoria at near than distance, a decreased AC/A ratio, and a decreased positive fusional vergence [7] and the convergence insufficiency symptom survey (CISS) questionnaire is the initial standardized and valid tool used to evaluate the frequency and nature of symptoms in patients with CI [8, 9]. Vision therapy is typically considered as the primary treatment option for CI, with base in prism prescription being an alternative choice [6]. However, given the age-related changes in the interaction and functioning of the accommodation and convergence systems, it is crucial to study how these systems interact in elderly individuals [7]. It is also important to compare the responses of these age groups to vision therapy and base in prism prescription [10]. While vision therapy has proven effective in reducing symptoms and enhancing visual function in patients with CI, the different accommodation and vergence systems in elderly individuals may yield different results [11]. There is little information about CI in presbyopic subjects, to our knowledge, in these patients, only one study investigated vision therapy [12] and base in prism prescription in which prism proved superior albeit in a very small sample of participants [13].; also patients age can impact the vision therapy compliance [14] and the documentation of effectiveness of vision therapy and base in prism prescription in presbyopic individuals with convergence insufficiency is insufficient.

## Methods/design

### Objectives of the study

The primary objective of this 3-parellel arm RCT is to assess the efficacy of home-based vision therapy and base in prism prescription in improvement of NPC and PFV, in presbyopic individuals who have CI. Three comparisons will be made: differentiation of the home vision therapy from base in prism prescription, and differentiation of home vision therapy from control group, differentiation of base in prism prescription from control group. Additionally, as secondary objectives, the study will involve a subjective evaluation of the clinical significance of any differences observed among groups and before and after of the interventions, using modified CISS questionnaire. We considered that a minimum variation of, 4 cm, 4 prism diopter, 6 prism diopter, and 6 points are necessary for clinically significant treatments for NPC, phoria, positive fusional vergence, and CISS score. In phase 1 we modified the CISS for elderly subjects. For this purpose, we added “Using your near optical correction” before each item and we assessed validity and reliability of the questionnaire using classical and modern analysis. Ten specialist experts in binocular vision were invited to evaluate the survey’s relevancy, clarity, simplicity, content validity ratio, and face validity. The modified CISS-P were completed twice by 50 presbyopic individuals with convergence insufficiency (CI) and 50 presbyopic individuals with normal binocular vision. Interclass correlation coefficient (ICC) for the instrument and each item were computed to assess test-retest reliability. Also we used the jMetrik software to conduct Rasch analysis. The CISS were adopted for presbyopic subjects and using ROC curve the best cut off point, sensitivity, specificity values were established for presbyopic subjects.

### Study design

The study will involve a randomized, prospective, double-blind clinical trial, with both the patient and evaluator being unaware of the treatment assignment. This protocol is according to SPIRIT-Outcomes 2022 Checklist (for combined completion of SPIRIT 2013 and SPIRIT- Outcomes 2022 items) [15].

### Methods: participants, and outcomes

#### Study setting

The Noordidegan eye clinic in Karaj will be the source of participants for this study. In phase one of this project, we modified and adapted the Persian version questionnaire used in a previous study that focused on young adults [16], and proceed to test its reliability and validity for subjects with presbyopia. A preliminary examination will be conducted by optometrist Z.K.R, while optometrist S.A will handle the interventions. Patients who meet the inclusion criteria will be invited to participate in the study after the initial examination. The purpose and details of the research will be explained to the patients, and their informed consent will be obtained.

#### Eligibility criteria

For inclusion in the study, participants must meet the following criteria: a CISS score exceeding the cut-off point established in phase 1 using our modified CISS, near and far best corrected visual acuity of 20/20, presence of CI, near exophoria being at least 4 prism diopters greater than distant exophoria, near point of convergence greater than 6 cm, inadequate near-positive fusion as defined by the Convergence Insufficiency Treatment Trial (CITT) study [17], and normal monocular accommodation amplitude as per the Hofstetter formula.

Participants will be excluded from the study if they meet any of the following criteria: dry eye, any form of strabismus or amblyopia, spherical refractive error ≥ 3.50 diopters, astigmatism ≥ 2.50 diopters, history of prism prescription, vision therapy undergone less than five years ago, history of strabismus or refractive error surgery, history of eye trauma, use of any ophthalmic or general medications or drugs that affect ocular accommodation, such as phenylephrine, anticholinergics (mydriatics), carbonic anhydrase inhibitors, antihistamines, morphine and its derivatives, antidepressants, amphetamine, and imipramine, presence of more than one prism of vertical phoria, any mechanical limitations in the eye muscles, ocular muscle paralysis, nystagmus, and any systemic or neurological diseases that affect binocular vision, such as diabetes, myasthenia gravis, Graves’ disease, and multiple sclerosis.

To ensure blinding, participants will be kept unaware of which group they have been assigned to. Those who express interest in participating in the study and meet the eligibility criteria will be asked to sign an informed consent form. The optometrist responsible for recruitment, Z.K.R, will carry out the preliminary evaluation after the participant’s score in the modified CISS questionnaire has been determined.

#### Intervention

Three groups will be considered for the intervention. The intervention will be executed by S.A. This individual will be the only one with access to the random allocation file and will administer the treatment based on each individual’s random number as specified by the computer program. To generate a list of random numbers, the website www.random.org will be utilized. Consecutive numbers generated by the website, will be assigned randomly to patients in each of the study groups. The optometrist will be blinded to the evaluation outcomes. Each group will receive the treatment assigned by randomization again.

#### Control group procedure

The control group patients will only receive new near glasses with appropriated addition power, as a standard treatment for presbyopia. Additionally, they will perform a form of exercise that involves random and aimless eye movements at home. This placebo eye exercises will be performed at a fixed distance and over a small area, (6 m) with minimal separation (2 cm) of adjacent stimuli to avoid any impact of accommodative and convergence changes with reduction in eye-to-stimulus distances or if inter-stimulus separation is too large. This exercise will not affect the convergence and accommodation system and will be carried out under the supervision of the optometrist.

#### Home vision therapy group procedure

The home vision therapy group will also receive new near glasses with appropriated addition power, as a standard treatment for presbyopia. In addition, they will be given vision therapy exercises with an instruction form of how and the duration of these exercise. These accommodation/ convergence training includes: Voluntary convergence, Bug on string, Eccentric Circles, Jumping vergence, Barrel card, chiastopic fusion, Brock string, push-up training. The patient will perform a variety of exercises during each session, which will occur three times a week for 20 min–10 min before noon and 10 min at night. These exercises must be documented on a specialized form for a duration of two months. The home exercises consist of eight vision therapy exercises, with two exercises to be completed before noon and two at night. Each exercise should be completed for five minutes, meaning that four different exercises should be performed each day. In the following session, the next four exercises should be done. The exercises should be recorded on the instruction form throughout this time. To improve adherence to intervention exercises will be monitored using video communication applications.

#### Prism prescription group procedure

The prism prescription group will be given the same treatment protocol and instructions as the control group. However, near prismatic glasses will be prescribed using the Sheard’s criterion and their appropriated addition power, and the base in prism will be rounded up and evenly distributed between both eyes. Sheard’s criterion is most popular guideline for prism prescription and it demonstrates optimal effectiveness when applied to individuals with exophoric conditions [18]. They will also be instructed to perform random and aimless eye movements, of which will be done at a distance of 6 m and over a 2 cm area, as mentioned previously they will not affect the convergence and accommodation system.

In all groups, if participants have a refractive error for distance vision, they will be provided with two separate pairs of spectacles, one for near use and one for distance use. To ensure regular usage of the near spectacles (with or without prism) throughout the study, adherence will be monitored through video calls and by participants confirming their usage on an instruction form.

#### Outcomes

The primary outcomes we focused on were the improvement in NPC and NPFV measures at break and recovery points, and the secondary outcomes were improvements in Convergence Insufficiency Symptom Survey (CISS) score, vergence facility, near heterophoria, stereopsis, prism adaptation, NNFV, that will be assessed before and after the intervention and comparison will be done among the groups. These primary and secondary goals are directly related to objective and subjective aspects of convergence insufficiency respectively.

#### Participant timeline

**Fig. 1:**
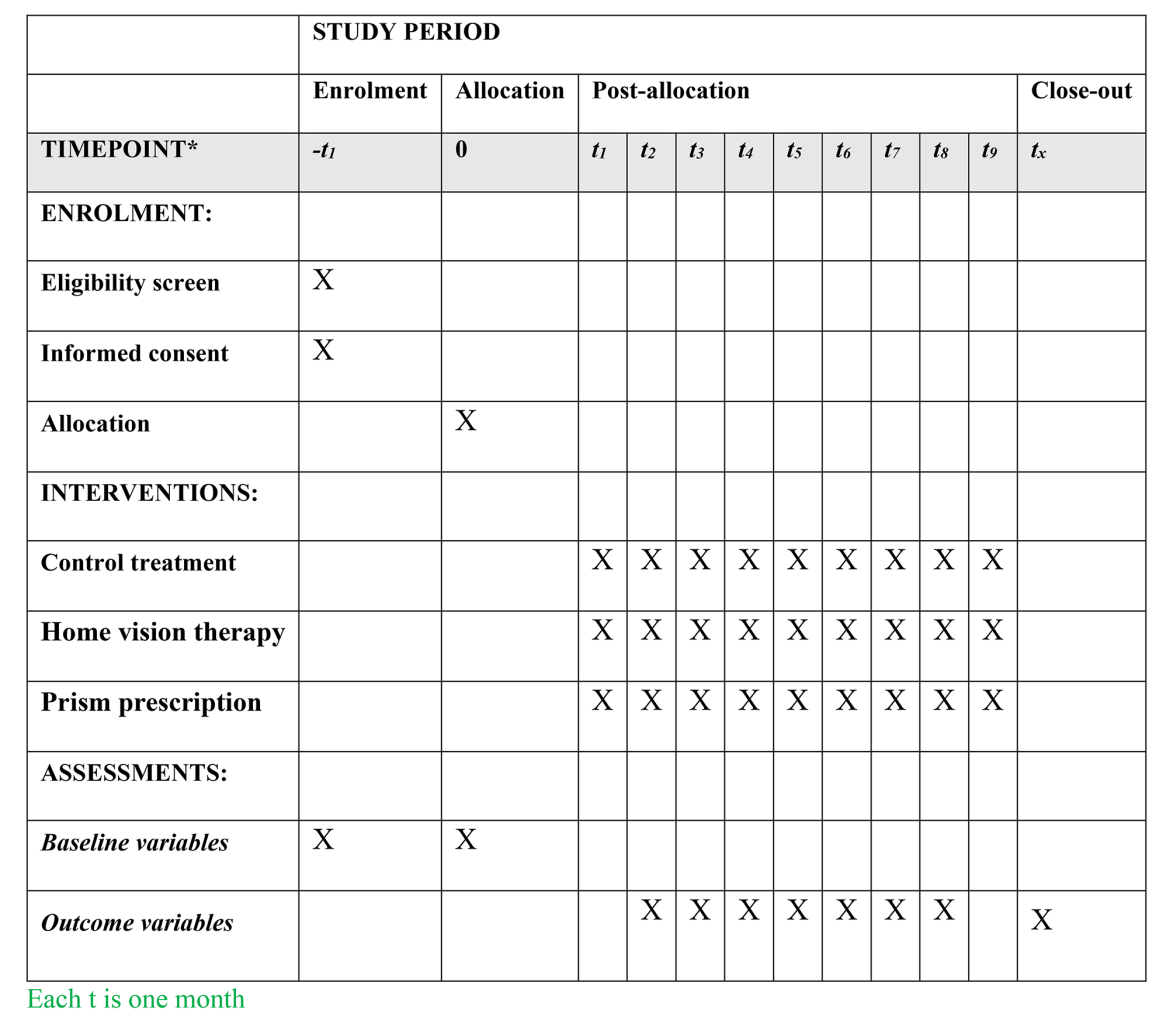
The predicted participant timeline of enrolment, interventions, and assessments

For all the groups after confirmation, all the prescribed glasses will be inspected and utilized, and the patients will not be aware of the group to which they belong. The vision therapy, aimless eye movements and utilization of the glasses will persist for a duration of two months. The follow ups will occur one and two months after the start of the treatment. Optometrist S.A will carry out all optometric exams once again. Figure 1 shows schedule of the study.

#### Safety assessment

Every unfavorable incident will be documented and sorted based on whether they are linked to the intervention. Any events that may be expected due to the assumed treatment mechanism, such as nausea, headache, and diplopia will be recorded. However, they have not been observed in previous studies.

**Fig. 2:**
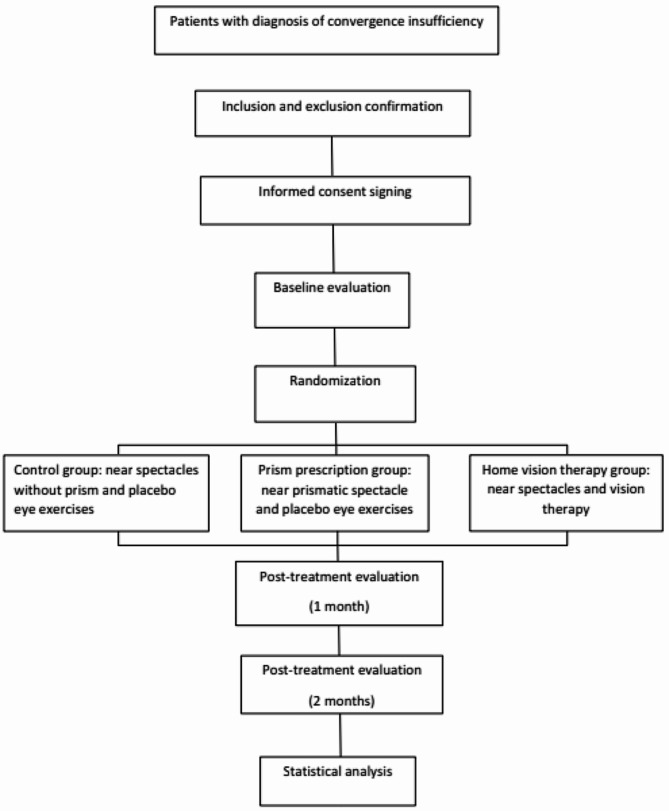
Flow chart of the study

Figure 2 provides a flowchart outlining various stages involved in of the study.

#### Sample size calculation

To calculate the appropriate sample size for all outcome variables specially those related to primary outcomes, a two-tailed test with α = 5% and β = 10% and 90% power was conducted using the Power Analysis & Sample Size software (PASS, version 11). The selection of appropriate statistics for the sample size determination was based on three previous studies [10, 12, 13]. The sample size in each group was determined using the One-way ANOVA menu of PASS 21 software, taking into account the quantitative nature of the dependent variables and the presence of three groups. The test was conducted with a first type error (α) of 0.05 and a second type error (β) of 0.1, corresponding to a test power of 90%. The mean values of 17 cm, 16 cm, and 21.52 cm, along with a standard deviation of 7.50, were extracted for NPC, and the effect size was calculated as 0.311. Considering a 15% dropout rate, the total number of samples in each group amounted to 50, resulting in a combined total of 150 samples.

### Sample recruitment

#### Methods: assignment of interventions (for controlled trials)

To allocate patients to the randomized treatment groups, the patients will be categorized based on their age, gender, and the severity of their CI using random blocks. We will have 3 groups: control group, prism prescription group, and home vision therapy group. To generate a list of random numbers, the website www.random.org will be utilized. Consecutive numbers generated by the website (ranging from 1 to 150) will be assigned randomly to patients in each of the group by optometrist Z.K.R., who has no prior knowledge of the random numbers list. The optometrist responsible for intervention, S.A., will be the only person who has access to the random numbers list. Due to the nature of vision therapy and other training instructions provided to the subjects, this optometrist will not be blinded. So participants get assigned consecutive numbers and then the randomized assigns treatments to each number. 1 optometrist applies the treatment. The other masked optometrist makes the measurements. Consequently, the allocation concealment will be ensured because the optometrist in charge of recruitment will be unaware of the group to which the subject will be assigned.

### Methods: data collection, management, and analysis

#### Data collection

To enhance data quality, measurements will be conducted twice during each session, and the modified questionnaires will undergo evaluation for their suitability among adults with presbyopia in terms of reliability and validity. This assessment will take place during the phase one of the study. In order to enhance participant retention and ensure successful follow-up, we will establish communication with the participants and provide encouragement to continue with the prescribed exercises. Moreover, in home vision therapy group, the patients are provided with a form to record the duration of their home exercises.

The optometrist responsible for recruitment in this study, Z.K.R. will conduct all the assessments. Throughout the study, Z.K.R. will maintain blindness regarding the group to which the subject has been assigned.

Table 1 displays the potential values and demographic variables of the subjects. The following variables will be recorded at the start of treatment: age, gender, distance best corrected visual acuity, near best corrected visual acuity, dry refraction, cycloplegic refraction, presence of dry eye, inter-pupillary distance and medications that may affect accommodation and vergence.

**Table 1:** Potential values and demographic variables

Variable	Values
Age	Years
Gender	Male/Female
Distance best corrected visual acuity	Log MAR
Near best corrected visual acuity	Log MAR
Dry refraction	Diopter
Cycloplegic refraction	Diopter
Dry eye	Yes/No
Medication	Yes/No
Inter-pupillary distance	Millimeter

MAR: minimum angle of resolution

Table 2 displays the measuring tool used, assessment time, and outcome variables. A single experienced optometrist (ZKR), who is also responsible for recruitment, will conduct all assessments. The optometrist conducting the assessment will be blinded to the group assigned to each participant.

**Table 2:** outcome variables

Outcome variables	Assessment time			Measuring tool
	Baseline	1 month post treatment	2 month post treatment	
Convergence insufficiency	*	*	*	CITT definition [17]
Vergence facility	*	*	*	Prism flipper
Distance heterophoria	*	*	*	Prism cover test
Near heterophoria	*	*	*	Prism cover test
Stereopsis	*	*	*	TNO test
Prism adaptation	*	*	*	Prism trial lens
Near positive fusional vergence (blur point)	*	*	*	Prism bar
Near positive fusional vergence (break point)	*	*	*	Prism bar
Near positive fusional vergence (recovery point)	*	*	*	Prism bar
Near negative fusional vergence (blur point)	*	*	*	Prism bar
Near negative fusional vergence (break point)	*	*	*	Prism bar
Near negative fusional vergence (recovery point)	*	*	*	Prism bar
Convergence insufficiency symptom survey score	*	*	*	Questionnaire
Near point of convergence with red green lenses (break)	*	*	*	Red-Green lenses and 500 mm ruler
Near point of convergence with red green lenses (recovery)	*	*	*	Red-Green lenses and 500 mm ruler
Near point of convergence with accommodative target (break)	*	*	*	Accommodative target and 500 mm ruler
Near point of convergence with accommodative target (recovery)	*	*	*	Accommodative target and 500 mm ruler

CITT: Convergence Insufficiency Treatment Trial

Vergence facility will be assessed using a flipper prism (3 base in and 12 base out), with the target set at two lines above the best near-corrected visual acuity (20/30). The test will be conducted with the patients near glasses on and recorded at a distance of 40 cm. The distance heterophoria will be assessed by means of prism bar and alternate cover test with the digital Snellen chart set at 20/30 raw with a single letter at a distance of 6 m. The near heterophoria will be assessed by means of prism bar and alternate cover test and over the patients near glasses with a 20/30 single optotype set at 40 cm. Stereopsis will be evaluated using the TNO test with red-green filters and the patients near glasses at 40 cm. Near negative fusional vergence, with a rate of 2 prisms per second, will be obtained using prism bar and a 20/30 single E chart set at 40 cm over patients near correction (in the blur, break and recovery points). Prism adaptation will be checked by measuring the amount of heterophoria one hour after wearing the glasses, and the percentage change in heterophoria will be recorded as prism adaptation. Near positive fusional vergence (in the blur, break, and recovery point) will be measured using a base-out prism bar with a 20/30 column set at 40 cm and the patients near glasses with a rate of 2 prisms per second. The modified CISS questionnaire will be used to assess the frequency and type of symptoms before and after treatment. The near point of convergence will be evaluated using the red-green filters and accommodative target methods. A penlight will be brought close to the patient’s face, and the patient will be asked to report the diplopic image as soon as possible. This point will be recorded as the break point. The examiner will then increase the distance between the penlight and the patient’s face until a single image reappears, which will be recorded as the recovery point. The near point of convergence will be measured three times, and the results will be averaged. The near point of convergence will be recorded based on both the break and recovery points, with the patient wearing their near glasses. In the accommodative target method, participants will be guided to maintain visual singularity while a single target with a 20/30 visual acuity will be gradually brought closer to them. The movement of the target will persist until diplopia is reported or the examiner detects an objective loss of binocular vision. The target will then be moved backward until either the participant subjectively reports single vision or the examiner objectively observes the restoration of binocularity.

To ensure that the optometrist conducting the clinical testing remains unaware of the prism near spectacles, in follow up visits, all glasses will be examined in a separate unit by another optometrist and S.A will not investigate the new habitual spectacles.

To ensure and encourage adherence, a written log detailing the daily usage of near spectacles, including approximate durations and descriptions of home exercises, will be maintained. In addition, video calls will be conducted to monitor and promote compliance. Participants are strongly encouraged to remain engaged in the study. Individuals who have successfully finished the study will receive the spectacle prescription at no cost. In the event that a participant is unable to continue or cooperate, they will be replaced by another individual.

#### Data management

To enhance data quality, a double-coding process will be employed for all participants, where all data will be coded independently and reviewed twice to ensure accuracy and consistency.

#### Statistical methods

Analysis will undergo using the Statistical Package for the Social Sciences software (SPSS version, 22). The significance levels of α and β will be set at 5% and 10%, respectively, while the confidence interval will be established at 95%. To compare the research variables before and after the intervention, the paired t-test or its non-parametric equivalent will be utilized. The normality of the data will be checked using the Kolmogorov- Smirnov test. One-way ANOVA with Tukey’s post hoc test will be employed to compare the quantitative variables in the study. ANCOVA will be used to eliminate any intervening variables. The Kruskal-Wallis and Mann-Whitney tests will be used to evaluate groups of quantitative variables in non-parametric conditions. For within-group analysis, repeated measures ANOVA will be conducted when initial homogeneity and normality of data are met. If initial homogeneity is established without normality of data distribution, the Friedman test will be used. A linear mixed model adjusted for the baseline variables will be employed when initial homogeneity is not found. Per protocol analysis principles will be applied in the study. Additionally, univariate analysis and multiple regression analysis will be conducted to identify the predictor factors that influence prism effectiveness in presbyopic patients.

#### Methods: data monitoring

The data monitoring committee (DMC) for this study will consist of S.A, Z.K.R, H.K, A.A.B, and S.R. As the trial data is collected, the DMC will review it for safety concerns, initial indications of treatment benefits or risks, and any indications of treatment ineffectiveness.

This study will be sponsored by Shahid Beheshti University of Medical Sciences, without any conflicts of interest, and does not involve interim analysis, study design, data collection, publications. During each examination session and through video calls, the patients will be asked about their level of success while performing exercises as well as any potential side effects they may be experiencing. In this trial auditing procedure will not be done.

## Discussion

This study represents the first randomized controlled trial that compares home vision therapy and base in prism prescription with a control group in the geriatric population affected by convergence insufficiency. Currently, there is limited data available on the management of convergence insufficiency in presbyopic individuals. The primary objective of this study is to assess whether the improvements in convergence insufficiency achieved through vision therapy and base in prism prescription, as observed in previous non-controlled studies, can be maintained under controlled clinical trial conditions. Additionally, the study aims to determine if there are any significant differences between home vision therapy and base in prism prescription, as well as between these two interventions and the control group. The ultimate goal is to identify the optimal treatment strategy for the development of effective management protocols. While a decrease in the CISS questionnaire score alone may not indicate successful treatment of CI, it is a clear indicator of a decrease in visual symptoms [18–20]. In patients with CI, vision therapy aims to improve the coordination and flexibility of the eyes, helping them work more effectively together to reduce symptom [21]. The exact mechanisms by which vision therapy improves CI are not fully understood, but it is thought to involve changes in the neural connections between the eyes and brain, as well as improvements in eye muscle coordination and visual processing [22]. However, in CI base in prism prescription decrease convergence demand in vergence system. prism glasses work by shifting the visual image slightly inward, which can help reduce the amount of effort required by the eyes to maintain binocular vision [23]. Overall, recent studies suggest that both vision therapy and base-in prism prescription can be effective in reducing CISS scores in patients with CI, with some studies suggesting that vision therapy may be more effective [18, 21, 24, 25]. Identifying the most effective treatment method for CI could be beneficial for managing symptoms in these patients.

Ensuring compliance is crucial for the effectiveness of home vision therapy treatment. Consequently, this study also seeks to evaluate the level of compliance among patients undergoing each treatment approach. Additionally, the study aims to investigate the potential occurrence of adverse effects associated with vision therapy or base in prism prescription. It has been hypothesized that there might be a theoretical risk of adverse events such as nausea, headache, diplopia, and prism adaptation. However, previous studies have not reported any instances of these events.

The implementation of masking procedures guarantees that clinical assessments are conducted in a manner that eliminates bias. Consequently, the treating optometrist will have no access to assessment results, either through the database or medical notes. Furthermore, strict controls are in place to prevent the treating optometrist from providing further treatment to the participants after the study, thereby ensuring that they cannot obtain the resultant binocular measurements.

The focus of this study is dedicated to patients aged 40 years and above. Birnbaum et al. [12] conducted a study involving patients aged over 40 years, which resulted in lower functional cure rate in home-based vision therapy than office-based vision therapy. This improvement was observed in terms of near point of convergence and positive fusional vergence development. In presbyopic patients with CI Wick [26] expressed that 10–15 weeks of home vision therapy can improve stereopsis and near point of convergence in 92% of patients. In a retrospective study by Westman et al. [27] in patients with age of 6–79 years of old ages office based vision therapy caused 37% improvement in near point of convergence and near positive fusional vergence. Also the findings of Teitelbaum et al. [13] demonstrated enhancements in individuals with convergence insufficiency (CI) who were also presbyopic, achieved through the application of progressive additional lenses with base in prism. Therefore, it is plausible that similar improvements could be attained in older patients utilizing base in prism prescription and vision therapy. However, presently there are no formal plans to evaluate this specific aspect, as the primary objective is to establish an optimal treatment protocol for individuals with CI who are also presbyopic.

The findings of this study will contribute to the advancement of future developments. It will be essential to establish an optimal treatment protocol, which may entail increased frequency of visits or a greater number of treatments to achieve a lasting impact. Additionally, long-term follow-up of patients will be necessary to ensure the sustained maintenance of beneficial effects. There is an immense possibility to develop home vision therapy and prism prescription as highly effective treatments for CI. This opens up various avenues for implementing these treatments in different settings such as hospital orthoptic clinics, prism prescription, and home-based vision therapy. As a result, there is a significant opportunity to enhance both vision and quality of life for older adults while also reducing treatment expenses.

### Trial status

As of 3 October 2023, 121 patients have been randomised. Recruitment will continue until all patients are randomised.

## Data Availability

Not applicable.

## Declarations

### Protocol amendments

Relevant authorities will receive timely notifications regarding any significant modifications made to the protocol.

### Dissemination policy

Our intention is to publish multiple articles highlighting various aspects of the study. However, we do not plan to grant public access to the underlying statistical data. Our future dissemination plan is to ensure that the study results reach the relevant scientific community, policymakers, healthcare professionals, and the general public, thereby contributing to the advancement of knowledge and potential implementation of findings in practice.

## Abbreviations

CI: Convergence insufficiency
NPC: Near point of convergence
PFV: Positive fusional vergence
CISS: Convergence insufficiency symptom survey
ICC: Interclass correlation coefficient
CITT: Convergence Insufficiency Treatment Trial
PASS: Power Analysis & Sample Size software
SPSS: Statistical Package for the Social Sciences software
DMC: Data monitoring committee
